# Emerging Threat of Multidrug Resistant Pathogens From Neonatal Sepsis

**DOI:** 10.3389/fcimb.2021.694093

**Published:** 2021-07-12

**Authors:** Hua Zou, Xiaojiong Jia, Xiao He, Yan Su, Ligang Zhou, Yan Shen, Chenglin Sheng, Ang Liao, Chunli Li, Qiuhong Li

**Affiliations:** ^1^ Department of Laboratory Medicine, Chongqing Health Center for Women and Children, Chongqing, China; ^2^ Department of Laboratory Medicine, First Affiliated Hospital of Chongqing Medical University, Chongqing, China; ^3^ Department of Neonatology, Chongqing Health Center for Women and Children, Chongqing, China; ^4^ Department of Laboratory Medicine, Wan Zhou Health Center for Women and Children, Chongqing, China; ^5^ Department of Laboratory Medicine, Yong Chuan Health Center for Women and Children, Chongqing, China

**Keywords:** neonates, multidrug-resistant, antimicrobial resistance, bloodstream infections, early-onset bloodstream infections, late-onset bloodstream infections

## Abstract

Multidrug-resistant (MDR) pathogens are responsible for a substantial burden of morbidity and mortality from neonatal sepsis; however, data on these sepsis-related pathogens among hospitalized neonates in China are not well characterized. In this study, a total of 240 strains were isolated from four Women and Children’s hospitals in Southwest China between 2014 and 2019. Of these included pathogens, 104 (43.33%) were gram-positive bacteria, 129 (53.75%) were gram-negative bacteria, and 7 (2.92%) were fungi. *Escherichia coli* (*E. coli*, 34.01%) and *Klebsiella pneumoniae* (*K. pneumoniae*, 15.35%) were the main pathogen of neonate bacteremia. ST167 were the most prevalent STs in *E. coli* and ST11 in *K. pneumoniae.* Our study found that *E. coli* (62.71%) was the predominate pathogen of early-onset sepsis, among which 64.86% were MDR. Late-onset sepsis was mainly caused by *K. pneumoniae* (28.31%) and *E. coli* (24.78%), with showing that 78.33% of these pathogens were MDR. Notably, the prevalence of EO/LO pathogens were quite different from Indian and south of China. Moreover, we found that *bla*_CTX-M_ (42.06%) was most dominant resistant genes with about a third isolates (31.09%) were positive for *bla*_CTX-M-15_. All the carbapenem-resistant *K. pneumoniae* were positive for NDM-1. Moreover, late-onset sepsis and antibiotic exposure were significantly associated with MDR infection. Emerging multi-resistant pathogens of sepsis posts a serious threat to neonatal outcomes and emphasizes an urgent need to control their further spread.

## Introduction

Premature birth, severe infection, and suffocation are the leading causes of neonatal death, with sepsis being the most frequent infection observed in neonatal wards  (Seale et al., 2013). Based on population-level studies during the last two decades, the global estimate of the incidence of neonatal sepsis was 2202 per100,000 live births (LBs), with mortality between 11% and 19% (Dong et al., 2020). Progress in neonatal survival, however, has lagged behind with global reductions in pediatric mortality, and neonatal deaths currently account for nearly half of all under-five deaths (Johnson et al., 2020).

Neonatal sepsis was divided into two groups according to the time of onset, early-onset sepsis (EOS) occurs within the first 3 days of life, whereas late-onset sepsis

(LOS) occurs after 4 to 7 days within the first 1 to 3 months of life. Group B Streptococcus (GBS) and *Escherichia coli* (*E. coli*) are predominant pathogens of neonatal EOS in four US States from 2005 to 2014 (Schrag et al., 2016), whereas gram-negative bacteria (GNB) like *E. coli* and coagulase-negative staphylococci (CoNS) were the leading pathogen in developing countries, such as China (Jiang et al., 2019). Infants with LOS were classified as community- or hospital-acquired LOS if onset of infection was 48 or >48 h following admission. According to the reports, GBS and *E. coli* were most prevalent community-acquired LOS, whereas CoNS, *Staphylococcus aureus*, and other GNB were mostly isolated in hospital-acquired LOS (Giannoni et al., 2018; Saha et al., 2018).

Appropriate treatment of sepsis empirically is crucial in reducing mortality. Nevertheless, as antimicrobial resistant (AMR) pathogens continue to emerge, the treatment of bacteremia becomes increasingly complicated. Most AMR pathogens exhibit high resistance to first-line drugs recommended by WHO, such as ampicillin, gentamicin, and cefotaxime, showing a multidrug-resistant (MDR) phenotype (Chaurasia et al., 2019). Especially MDR in Gram-negative bacteria that are the greatest concern in the neonatal population, with a worldwide rise in the reported incidence. A recent meta-analysis of Chinese literature (2009–2014) on neonatal sepsis revealed that more than 50% of *E. coli* and *Klebsiella* spp. were resistant to third-generation cephalosporin, and the resistance of ampicillin in *E. coli* was almost 80% (Li et al., 2018), similar to that in the US (Stoll et al., 2011). β-lactamase was one of most important resistant mechanism of MDR, which could combine with the β-lactams antibiotics, break the amide bond of the four-membered azetidinone ring present in every β-lactam, then eliminate the killing activity (Tooke et al., 2019). According to the structure and function, β-lactamases were usually divided into four classes, including class A (such as TEM, SHV, CTXM, and KPC), C (Ampc), and D (OXA) enzymes, with a serine in the active site, and class B (such as IMP, NDM, and VIM), metallo-β-lactamase (Bush and Jacoby, 2010). Significantly, β-lactamases could be produced through mutations in chromosomal genes or by acquisition of foreign DNA encoding new β-lactamases leading to a worldwide pandemic (Eichenberger and Thaden, 2019). What’s more, it had reported that MDR especially producing β-lactamase with very limited therapeutic options resulted higher mortality (Johnson et al., 2020). However, to date, information regarding the neonatal sepsis and clinical characteristics of MDR in China remains elusive. Better understanding of these data may enable early recognition of patients at high risk and potentially reduce the burden of neonatal sepsis and MDR in clinical settings.

## Materials and Methods

### Study Design and Patients Collection

We conducted a retrospectively cohort study from January 2014 to December 2019 from Neonatal ward in four different hospitals: Chongqing Health Center for Women and Children, Wanzhou Health Center for Women and Children, Yongchuan Health Center for Women as well as Children and Northern Maternity Hospital in China. There were two general Neonatal wards with a combined bed capacity of 80, including 25 incubators, 40 small beds, 15 cots, and two Neonatal ICU ward with a bed capacity of 20 in Chongqing Health Center for Women and Children. Wanzhou Health Center for Women and Children, Yongchuan Health Center for Women has two neonatal wards and each contains 48 beds. Children and Northern Maternity Hospital has only one neonatal ward with 25 beds. Blood cultures were performed on suspected sepsis patients in the same manner with Children’s blood culture bottles contained antibiotic adsorbents, such as resin. Finally, patients with clinical features consistent with blood infection were included in the analyses.

### Inclusion and Exclusion Criteria

To fully understand the pathogenic bacteria of neonatal sepsis, we formulated detailed inclusion criteria and exclusion criteria as follows. Inclusion: newborns had at least one positive blood culture and corresponding clinical manifestations, such as fever, poor response, poor feeding, edema, jaundice, abdominal distension, vomiting, dyspnea and apnea, cyanosis, pale complexion, cold limbs, tachycardia, bradycardia, marbly-like skin pattern, hypotension, or capillary filling time >3 s (Shane et al., 2017). Exclusion: age > 28 days. The positive blood culture was not consistent with the clinical manifestations, which was suspected to be contaminated bacteria. For patients with more than 1 episode of sepsis, we only considered the first episode.

### Bacterial Strains

All these non-repetitive isolates were identified at the species level by the VITEK MS (bioMerieux, Hazelwood, MO, United States) system, and routine antimicrobial susceptibility testing was performed by the VITEK2 compact (bioMérieux, Inc., Durham, NC) system. According to the recommended guidelines of Clinical and Laboratory Standards Institute (CLSI, M100-S27) (Institute, 2020), the penicillin (P), ceftazidime (CAZ), ceftriaxone (CRO), cefepime (FEP), ertapenem (ETP), imipenem (IPM), meropenem (MEM), levofloxacin (LEV), ciprofloxacin (CIP), amikacin (AMK), gentamicin (GEN), vancomycin (VAN), teicoplanin (TEC), and linezolid (LZD) were determined using the broth microdilution method. At the same time, *E. coli* ATCC25922 was selected as the standard strains for MIC detection. All standard strains were purchased from American Type Culture Collection and all antibiotics were purchased from Meilunbio in China.

### DNA Amplification and Analysis

All *E. coli* and *K. pneumoniae* were selected to detect drug resistant genes. Total DNA was extracted by boiling. Briefly, single colonies were picked from overnight culture of each isolate, resuspended in 200 ul of sterile distilled water, and boiled at 100°C for 10 min. After centrifugation at 15,000*g* for 15 min, supernatants were collected and stored at −20°C (Jia et al., 2018). Polymerase chain reaction (PCR) was used to detect the potential presence of carbapenemase genes, including *bla*_KPC_, *bla*_NDM_, *bla*_VIM_, *bla*_IMP_, and *bla*_OXA−48_, and sequencing was used to confirm the variants of these carbapenemase genes (Jia et al., 2018). Moreover, ESBLs, such as *bla*_CTXM_, *bla*_TEM_, *bla*_SHV_, and *bla*_OXA-1_, AmpC, such as *bla*_ACC_, *bla*_FOX_, *bla*_MOX_, *bla*_DHA_, *bla*_CIT_, and *bla*_EBC_, porin genes, OmpF, and OmpC, aminoglycoside (*aac(3)-II. aac(6’)-Ib, ant(3”)-I, and armA, rmtB*), and fluoroquinolone resistancegenes (*qnrA*, *qnrB, qnrC, qnrD*, and *qnrS*) were also determined by using primers as described previously (Tian et al., 2018). *E. coli* E001 isolated from clinical, which were positive for *bla*_NDM-1_, *bla*_KPC-2_, *bla*_CTX-M15,_ and *bla*_SHV-1_ as the standard strains. All PCR-related reagents, such as PCR enzymes (TaKaRa Taq Cat No R001A), were purchased from Takara Bio. The primers were synthesized by Shanghai Sangon Company and the amplified products were also sent to Shanghai Sangon for sequencing.

### Multi-Locus Sequence Typing (MLST)

All *E. coli* and *K. pneumoniae* were selected to MLST. Multi-locus sequence typing (MLST) was performed by the amplifications of the internal fragments of seven housekeeping genes of *E. coli* isolates (*adk*, *fumC*, *icd*, *purA*, *gyrB*, *recA*, and *mdh*) and *K. pneumoniae* (*rpaB*, *gapA*, *mdh*, *pgi*, *phoE*, *infB*, and *tonB*) according to the database (https://pubmlst.org/organisms).

### Risk Factors and Clinical Outcomes of Neonate Suffered Sepsis

We conducted a retrospective case–control study to evaluate the risk factors and clinical outcomes of neonate with sepsis. Patients from 2014 to 2016 were exclude, because much information missed. Patients who suffered sepsis with complete medical records were selected as cases. Controls were identified as non-sepsis patients admitted into neonatal ward and excluded if with pneumonia, meningitis, and other infections.

### Definitions

Sepsis was diagnosed according to the criteria of the Centers for Disease Control and Prevention of bacterial isolation from the patient’s sterile body fluids accompanied by inflammatory response syndrome (Garner et al., 1988). AMR and MDR were acceding to the criteria of the Clinical and Laboratory Standards Institute 2020 (Institute, 2020). For gram-positive bacteria, we defined methicillin-resistant *Staphylococcus aureus* (MRSA), vancomycin-resistant enterococci (VRE) as MDR pathogens. For gram-negative bacteria, MDR were defined as bacteria were resistant to at least three antimicrobial classes. Extended-spectrum-β-lactamase (ESBLs) producing organisms were tested by the phenotypic tests of ESBL production (Weinstein, 2021). At the same time, *E. coli* ATCC25922 was selected as the standard strains for ESBL detection. Coagulase-negative Staphylococcus (CNS) infection was defined as: (1) Patients had infection-related symptoms, such as elevated body temperature and increased infectious markers on the blood culture day; (2) Multiple blood cultures were all CNS; (3) Catheter blood culture, peripheral blood culture or (and) catheter tip culture was CNS (Hurley, 2020).

### Statistical Analysis

All analyses were performed using SPSS v.25.0 software (SPSS Inc., Chicago, IL, USA). Univariate analyses were performed separately for each of the variables. All variables with a P value of ≤0.05 in the univariate analyses were considered for inclusion in the multivariate logistic regression model. Ordered logistic analysis was used for the ordered multiple categorical variables. The odds ratio (OR) and 95% confidence interval (CI) were calculated to evaluate the strength of any association. Continuous variables were calculated using Student *t* test (normally distributed variables) and Wilcoxon rank-sum test (non-normally distributed variables) as appropriate. For all calculations, statistical significance was defined at P <0.05 for 2-tailed tests.

### Ethics Approval and Consent to Participate

For this study, samples were collected at the microbiology laboratory of our hospital, with no contact with the patient. This study was retrospective and there was no patient identification performed during data collection. Therefore, the ethics committee determined that informed consent was not required.

## Results

### Maternal and Neonatal Characteristics and Clinical Features

**Figure 1:**
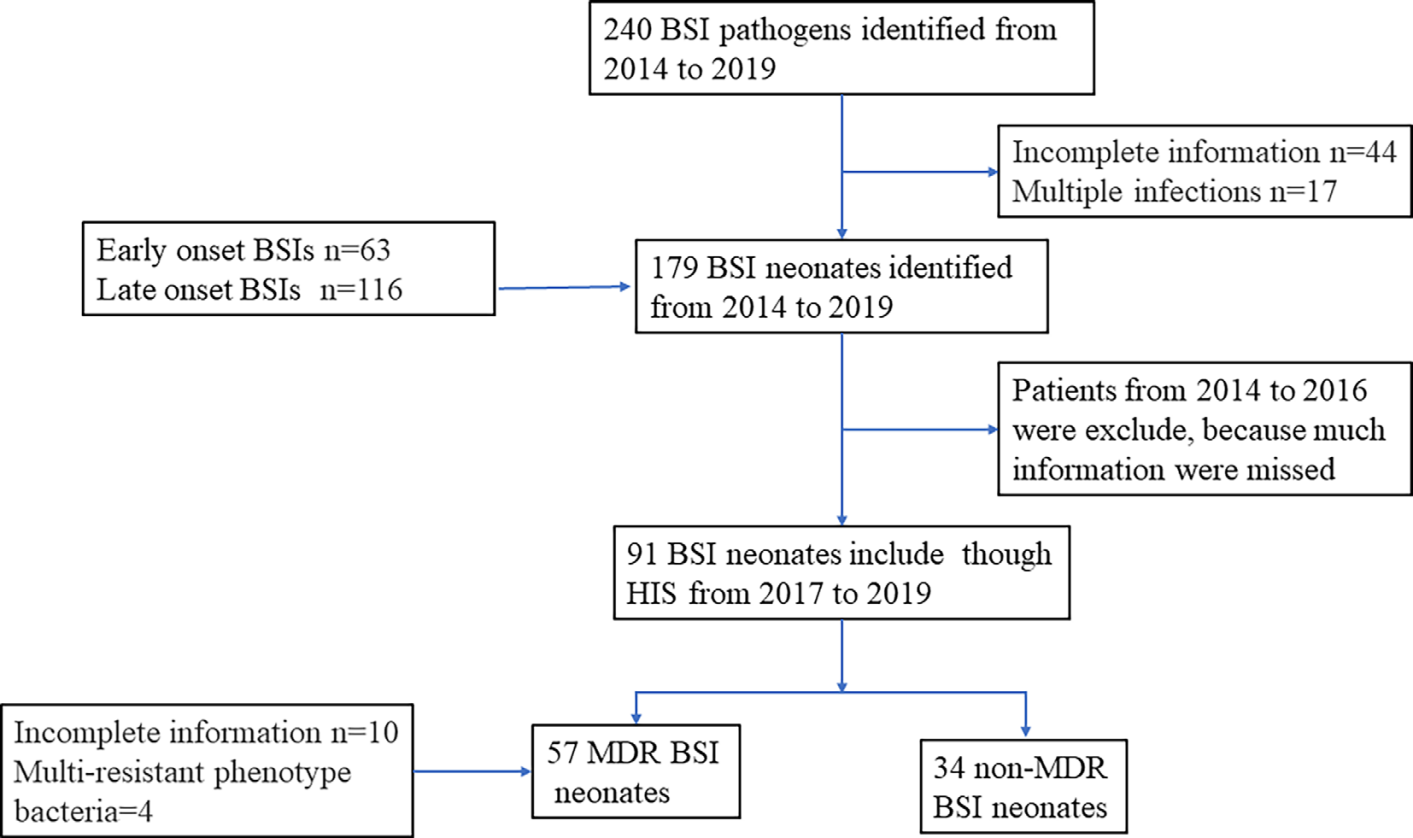
Flow diagram of the study process.

240 sepsis occurred from January 1, 2014, to December 31, 2019. 63 newborns developed EOS and 116 developed LOS when excluded patients with incomplete data and multiple infections. To collect more detailed and accurate clinical data, neonatal sepsis from 2014 to 2016 was excluded, and the last 91 neonatal sepsis cases with complete cases were collected (**Figure 1**). As shown in **Table 1**, for neonates, 59.8% of the patients were male and the mean birthweight of enrolled neonates was 2517.8g (SD=934.8) among which 39.67% of the neonates were low weight newborn, 21.22% were very low weight newborn, and 10.05% were extremely low birth weight newborn. The mean gestation was 35.68 weeks (SD=4.2), of which preterm, very preterm, and extremely preterm account for 59.22%, 15.08%, and 10.05%, respectively. More than half of enrolled neonates (51.4%) were born by caesarean section. In addition, 14.5% of neonates were positive for embryonic membrane culture. Although 13.4% mothers received antibiotics within 7 days before delivery, less than half (5.6%) received antibiotics longer than 48 h. Among all inflammatory indicators, the sensitivity of WBC was the worst, with only 37.45% newborns greater than 20 × 10^9^/L or lower than 5 × 10^9^/L. Neutrophils and CRP increased in about half of the patients. Although the sensitivity of PCT was much higher, about three quarters of patients (77.7%) had increased after the occurrence of sepsis. When neonatal sepsis occurred, thrombocytopenia occurred in only 19.55% of patients (**Table 1**).

**Table 1:** Demographic details of enrolled neonates.

Variables		Number of neonates
Sex		
Boys		107 (59.8%)
Girls		72 (41.2%)
Age, days		9.2 ± 7.6
Birthweight, g		2517.8 ± 934.8
Weight of infants	macrosomia (>4000g)	5 (2.80%)
	normal weight newborn (≥2,500 g, ≤4,000 g)	47 (26.25%)
	low birth weight newborn (<2500g)	71 (39.67%)
	very low birth weight newborn (<1500g)	38 (21.22%)
	extremely low birth weight newborn(<1000g)	18 (10.05%)
Gestation, weeks		35.7 ± 4.2
Delivery gestational age	post term Infant (≥42 weeks)	3 (1.67%)
	Term Infant (≥37 weeks, <42 weeks)	25 (13.96%)
	preterm (≥32 weeks, <37 weeks)	106 (59.22%)
	very preterm (≥28 weeks, <32 weeks)	27 (15.08%)
	Extremely preterm (<28 weeks)	18 (10.05%)
Positive Culture of Embryonic membrane		26 (14.5%)
**Maternal and perinatal variables**		
Antenatal corticosteroids		31 (17.3%)
Maternal fever within 7 days before delivery		20 (11.2%)
Maternal antibiotics within 7 days before delivery		24 (13.4%)
Received for 48 h or more		10 (5.6%)
Caesarean delivery		92 (51.4%)
**Pathogens**		
Gram positive bacteria		99 (47.1%)
Gram-negative bacterium		105 (50.0%)
Fungus		6 (2.9%)
**Inflammatory biomarkers**		
WBC (>20*10^9/L or <5*10^9/L)		67 (37.43%)
Neutrophils% (>60%)		104 (58.10%)
Platelet (<100*10^9/L)		35 (19.55%)
CRP (neonates>0.6mg/L, Three days after birth>1.6mg/L)		102 (57.0%)
PCT (neonates >0.55 ng/ml, 12–24 h>4.7 ng/ml, 36–48 h>1.7 ng/ml, Three days after birth>0.05 ng/ml)		139 (77.7%)

### Pathogens Isolated from Neonates With Sepsis

**Figure 2:**
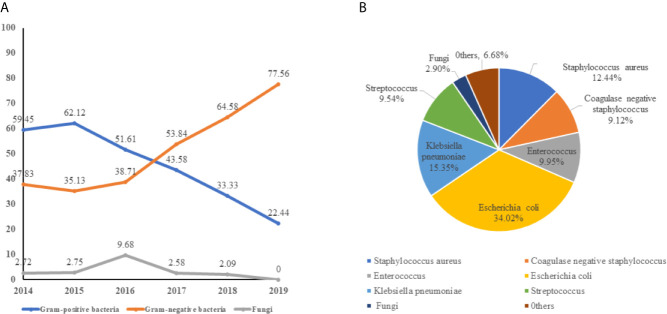
The distribution of pathogen of neonatal bloodstream infection. **(A)** the trend of pathogen of neonatal bloodstream infection from 2014 to 2019. **(B)** the composition of bacteremia pathogens.

Of all 240 pathogens, gram-positive bacteria, gram-negative bacteria, and fungi accounted for 104(43.33%), 129 (53.75%), and 7 (2.92%), respectively. Gram-negative bacteremia were the main pathogen and the isolation rate increased year by year, while the rate of gram-positive bacteremia decreases from 2016. Fungus (2.92%) is the rarest pathogen of neonatal sepsis (**Figure 2A**). It is worth noting that *E. coli* (34.01%) is the main pathogen of neonate bacteremia, followed by *K. pneumoniae*, accounted for 15.35%, other gram- negative bacteremia included *Enterobacter cloacae* (2.08%), *Acinetobacter baumannii* (1.25%), and *Enterobacter aerogenes* (0.83%) are less common in neonatal sepsis. For gram-positive bacteremia, *Staphylococcus aureus* (12.44%), *Enterococcus* spp. (9.95%), and *Streptococcus* spp. (9.54%) also played a significant role in sepsis (**Figure 2B**).

### Antimicrobial Susceptibility of Pathogens Isolated From Neonates

For the antimicrobial susceptibility of these pathogens from neonates, our study showed the highest resistant rate to cephalosporins especially in *K. pneumoniae, accounting for* 75.67%, whereas 43.92% of *E. coli* were resistant to Ceftriaxone. More than a quarter of the pathogens were resistant to quinolones and gentamycin. In addition, we found that about 13.51% of *K. pneumoniae* were resistant to carbapenems. However, these isolates were mostly susceptible to amikacin (**Table 2**). For Gram-positive bacteria, first, all staphylococcus were resistant to penicillin, whereas the rate of Enterococcus was as high as 66.67%. However, all group B streptococcus were susceptible to penicillin. Almost all of CNS were resistant to methicillin, whereas 53.33% *S. aureus* were methicillin-resistant *Staphylococcus* (MRSA). Second, more than two-thirds of staphylococci were resistant to clindamycin and erythromycin and about 40% resistant to levofloxacin. Finally, all gram-positive cocci were sensitive to vancomycin, teicolanin, and linezolid (**Table 3**).

**Table 2:** Antimicrobial susceptibility of Gram-negative bacteria isolates from neonate.

Antibiotics	*K. pneumoniae* (n = 37)			*E. coli* (n = 82)			Other pathogens (n = 10)		
	MIC_50_	Range	R, no. (%)	MIC_50_	Range	R, no. (%)	MIC_50_	Range	R, no. (%)
Ceftriaxone	64	2–512	28 (75.67)	2	0.25–64	36 (43.92)	0.5	0.5–8	1 (10.00)
Ceftazidime	64	1–512	26 (70.27)	1	0.25–64	14 (17.07)	0.25	0.25–32	1 (10.00)
Cefepime	32	0.25–512	25 (67.57)	1	0.25–64	22 (26.83)	0.25	0.25–32	1 (10.00)
Ciprofloxacin	0.5	0.25–16	11 (29.72)	0.5	0.25–16	24 (29.27)	0.5	0.25–1	0 (0.00)
Levofloxacin	0.5	0.25–16	9 (24.32)	0.5	0.25–16	22 (26.83)	0.5	0.25–1	0 (0.00)
Gentamycin	0.25	0.25–32	14 (37.84)	0.25	0.25–32	18 (21.95)	0.25	0.25–1	0 (0.00)
Amikacin	0.25	0.25–2	0 (0.00)	0.125	0.125–2	0 (0.00)	0.125	0.125–1	0 (0.00)
Imipenem	1	0.25–512	5 (13.51)	0.25	0.25–1	0 (0.00)	0.25	0.25–1	0 (0.00)
Meropenem	0.25	0.25–128	5 (13.51)	0.125	0.125–1	0 (0.00)	0.125	0.125–1	0 (0.00)
Ertapenem	1	0.25–512	5 (13.51)	0.5	0.25–1	0 (0.00)	0.25	0.25–1	0 (0.00)

**Table 3:** Antimicrobial susceptibility of Gram-positive bacteria isolates from neonate.

Antibiotics	*Staphylococcus aureus* (n=30)			CNS (n=23)			*Enterococcus spp* (n=24)		
	MIC_50_	Range	R, no. (%)	MIC_50_	Range	R, no. (%)	MIC_50_	Range	R, no. (%)
Penicillin	64	64–512	30 (100.00)	128	64–512	23 (100.00)	32	0.125–64	16 (66.67)
Methicillin	8	0.125–16	16 (53.33)	16	0.25–32	21(91.30)			–
Gentamicin	8	0.5–32	10 (33.33)	32	0.5–128	12 (52.17)	–	–	24 (100.00)
Vancomycin	0.25	0.125–1	0 (0.00)	0.25	0.25–2	0 (0.00)	0.25	0.125–1	0 (0.00)
Teicoplanin	2	0.5–4	0 (0.00)	4	0.5–4	0 (0.00)	2	0.5–4	0 (0.00)
Linezolid	1	0.25–2	0 (0.00)	1	0.25–4	0 (0.00)	1	0.25–2	0 (0.00)
Levofloxacin	8	0.25–16	12 (40.00)	8	0.25–16	10 (43.47)	0.5	0.25–2	0 (0.00)
Clindamycin	8	0.25–16	20 (66.66)	8	0.5–32	18 (78.26)	–	–	–
Erythromycin	16	0.25–32	22 (73.33)	16	0.25–32	18 (78.26)	0.5	0.25–16	5 (20.83)

### Genotypic Distribution of Pathogens Isolated From Neonates

**Figure 3:**
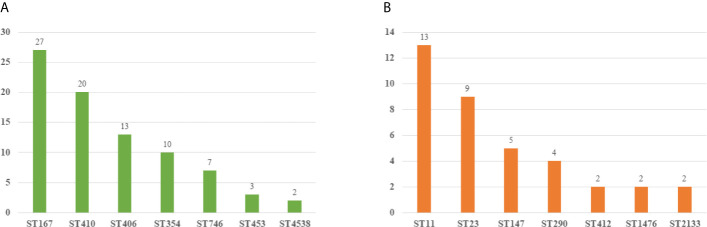
Molecular epidemiology of *E*. *coli* and *K pneumoniae.* **(A)** MLST types of *E*. *coli.* **(B)** MLST types of *K pneumoniae*.

We collected 119 isolates including 82 *E. coli* isolates and 37 K*. pneumoniae* isolates. Resistance genes and MLST in these strains were detected by PCR. We found that ST167 (27/82, 32.93%), ST410 (20/82, 24.39%), and ST406 (13/82, 15.85%) were the most prevalent STs in *E. coli*, whereas ST354 (10/82, 12.20%), ST746(7/82, 8.54%), ST453(3/82, 3.65%), and ST 4538 (2/82, 2.44%) were quite few. ST11 (13/37, 35.13%), ST23(9/37, 24.32%), and ST147(5/37, 12.51%) were most common in *K. pneumoniae*, other STs included ST290(4/37, 10.81%), ST412 (2/37, 5.41%), ST1476(2/37, 5.41%), and ST2133(2/37, 5.41%) were less common (**Figure 3**). As for resistant genes, CTX-M-15 (32.93%) was most dominant in *E. coli*, whereas the positive rate of SHV (15.85%) and TEM (10.97%) were low. SHV-2 (51.35%) was most prevalent in *K. pneumoniae*, followed by CTXM-9 (48.64%). All the carbapenem-resistant *K. pneumoniae* were positive for NDM-1. In addition, nearly a third of the isolates were positive for qnrB, whereas the positive rate of Ampc genes (1.68%) and Aminoglycoside (7.56%) resistance genes were quiet low (**Table 4**).

**Table 4:** Antibiotic resistance genes of *E. coli* and *K. pneumonia*.

Characteristics			Total (n=119)	*E. coli* (n=82)	*K. pneumoniae* (n=37)
**Carbapenemase type(s)**	**NDM-1**		5 (4.21%)	0 (0.00%)	5 (13.51%)
**ESBL type(s)**	**CTX-M**	CTX-M-15	37 (31.09%)	27 (32.93%)	10 (27.02%)
		CTX-M-14	22 (18.48%)	15 (18.29%)	7 (18.91%)
		CTX-M-9	26 (21.84%)	8 (9.75%)	18 (48.64%)
	**SHV-2**		22 (18.48%)	13 (15.85%)	19 (51.35%)
	**TEM-1**		21 (17.64%)	9 (10.97%)	11 (29.73%)
**AmpC genes**	**CIT**		2 (1.68%)	0 (0.00%)	2 (5.41%)
**Aminoglycoside resistance genes**	**aac(3)-II**		9 (7.56%)	7 (8.54%)	2 (5.41%)
	**aac(6’)-Ib **		5 (4.20%)	4 (4.88%)	1 (2.70%)
	**ant(3”)-I **		4 (3.36%)	4 (4.88%)	NA
	**armA**		3 (2.56%)	2 (2.44%)	NA
	**rmtB**		2 (1.68%)	2 (2.44%)	NA
**Fluoroquinolone resistance genes**	**qnrA**		13 (10.92%)	9 (10.98%)	4 (10.81%)
	**qnrB**		35 (29.41%)	24 (29.26%)	11 (29.73%)
	**qnrD**		10 (8.40%)	2 (2.44%)	8 (21.62%)
	**qnrS**		11 (9.24%)	2 (2.44%)	9 (24.32%)

NA, Not Applicable.

### Resistance Patterns for Early- and Late-Onset Pathogen

**Figure 4:**
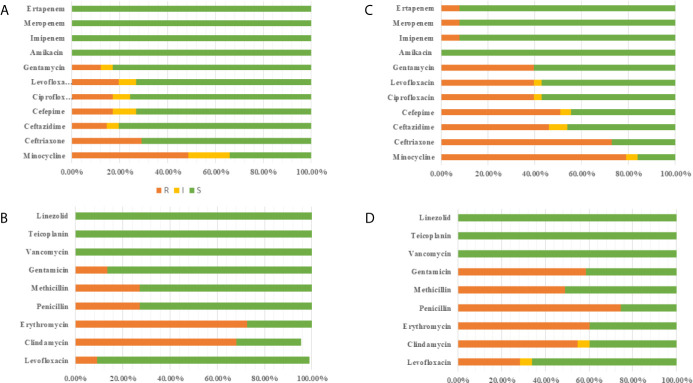
Antimicrobial susceptibility of pathogen in EOS and LOS. **(A)** Antimicrobial susceptibility of Gram-negative bacilli in EOS. **(B)** Antimicrobial susceptibility of Gram-positive bacilli in EOS. **(C)** Antimicrobial susceptibility of Gram-negative bacilli in LOS **(D)** Antimicrobial susceptibility of Gram-positive bacilli in LOS.

Excluding patients with incomplete data and multiple infections, 63 newborns developed early onset sepsis including and 116 late-onset sepsis from 2014 to 2017. **Figure 4** showed distribution of pathogens and resistance patterns between the early- and late-onset sepsis groups. For early-onset sepsis, gram-negative bacilli, and gram-positive cocci accounted for 65.08% and 34.92%, respectively. Gram-negative bacilli showed the highest rate of resistance to minocycline (48.78%), followed by ceftriaxone (29.25%), and levofloxacin (19.51%), while most sensitive to carbapenem (100.00%). 72.72% and 68.18% of gram-positive bacteria were resistant to erythromycin and clindamycin, only 13.63% and 9.09% of the bacteria were resistant to levofloxacin and gentamicin, and all the bacteria were sensitive to vancomycin, teicolanin, and linezolid.

The predominated pathogens were *E. coli* and Streptococcus spp. in EOS, accounting for 62.71% and 23.72%, respectively. *E. coli* was more resistant to cephalosporin (70.72%) but more sensitive to quinolone (18.91%), aminoglycosides (13.51%), and β-lactam/enzyme inhibitors (10.81%). 32.43% of the isolates produced ESBLs and none of the isolates produced carbapenemases. Importantly, 64.86% of *E. coli* were MDR in early-onset sepsis. All the Streptococcus spp were susceptible to ampicillin with 68.75% resistant to erythromycin. The pathogenic bacteria of late-onset sepsis contained 46.90% Gram-positive bacteria and 53.10% Gram-negative bacteria. Gram-negative bacteria were not only highly resistant to minocycline (79.19%), ceftriaxone (73.01%), cefepime (50.79%), and ceftazidme (46.03%), but also about 40.00% resistant to quinolone and gentamicin. To make matters worse, carbapenem resistant bacteria (7.93%) also appeared. In terms of gram-positive bacteria, 74.51% were resistant to penicillin, whereas 49.05% were resistant to methicillin. The resistance rate to gentamicin (58.49%) was also very high. Fortunately, all the bacteria were still sensitive to vancomycin, teicolanin, and linezolid.

*K. pneumoniae* (28.31%), *E. coli* (24.78%), and *S. aureus* (17.69%) the dominant pathogen in LOS. Notably, 85.71% of *E. coli* and 84.38% *K. pneumoniae* were resistant to cephalosporin with 76.66% producing ESBLs. What’s more 15.60% of *K. pneumoniae* produced carbapenemases, which were higher than early-onset sepsis. At the same time, more than 40% of *E. coli* were resistant to quinolones, aminoglycosides, and β-lactam/enzyme inhibitors, and *K. pneumoniae* was more resistant to quinolones (34.37%) and aminoglycosides (37.50%) but more sensitive to β-lactam/enzyme inhibitors (15.63%). What’s more, 78.33% of *K. pneumoniae* and 75.00% of *E. coli* were MDR in late-onset sepsis. In addition, 55.00% *S. aureus* were MARSA from late-onset sepsis (**Table 5**).

**Table 5:** Pathogen and drug susceptibility of early onset sepsis and late-onset sepsis.

Variable	Total	Early onset sepsis (n=63)	Late-onset sepsis (n=116)
Gram negative			
*E. coli*			
First or second generation cephalosporins	76.92% (50/65)	70.27% (26/37)	85.71% (24/28)
ESBLs	49.23% (32/65)	32.43% (12/37)	71.43% (20/28)
Carbapenems	0 (0/65)	0 (0/37)	0 (0/28)
Fluoroquinolones	33.84% (22/65)	18.91% (7/37)	53.57% (15/28)
Aminoglycosides	27.69% (18/65)	13.51% (5/37)	46.43% (13/28)
β-lactam/enzyme inhibitors	18.46% (16/65)	10.81% (4/37)	42.85% (12/28)
Tetracycline	63.07% (41/65)	54.05% (20/37)	75.00% (21/28)
MDR	69.23% (45/65)	64.86% (24/37)	75.00% (21/28)
*K. pneumonia*			
First or second generation cephalosporins	84.38% (27/32)	0	84.38% (27/32)
ESBLs	81.25% (26/32)	0	81.25% (26/32)
Carbapenems	15.6% (5/32)	0	15.6% (5/32)
Fluoroquinolones	34.37% (11/32)	0	34.37% (11/32)
Aminoglycosides	37.50% (12/32)	0	37.50% (12/32)
β-lactam/enzyme inhibitors	15.63% (5/32)	0	15.63% (5/32)
Tetracycline	84.38% (27/32)	0	84.38% (27/32)
MDR	81.25% (26/32)	0	81.25% (26/32)
**Gram positive**			
Coagulase-negative staphylococci			
Methicillin	100.00% (21/21)	100.00% (6/6)	100.00% (15/15)
Vancomycin	0 (0/21)	0 (0/6)	0 (0/15)
*Staphylococcus aureus*			
Methicillin	50.00% (11/22)	0 (0/2)	55.00% (11/20)
Vancomycin	0 (0/22)	0 (0/2)	0 (0/20)
*Enterococcus* spp			
Ampicillin	75.00% (12/16)	0	75.00% (12/16)
Vancomycin	0 (0/16)	0	0 (0/16)
*Streptococcus* spp			
Penicillin	0 (0/16)	0 (0/14)	0 (0/2)
Erythrocin	68.75% (11/16)	71.43% (10/14)	50.00% (1/2)

ESBLs, extended broad spectrum β-lactamase; MDR, multidrug resistance (ie, I [intermediate] or R [resistant] to on drug in three of the following classes: cephalosporins including first and second generation cephalosporins, fluoroquinolones, aminoglycosides, carbapenems, tetracycline, and piperacillin-tazobactam).

### Clinical Outcome and Risk Factors for Neonatal Sepsis

**Figure 5:**
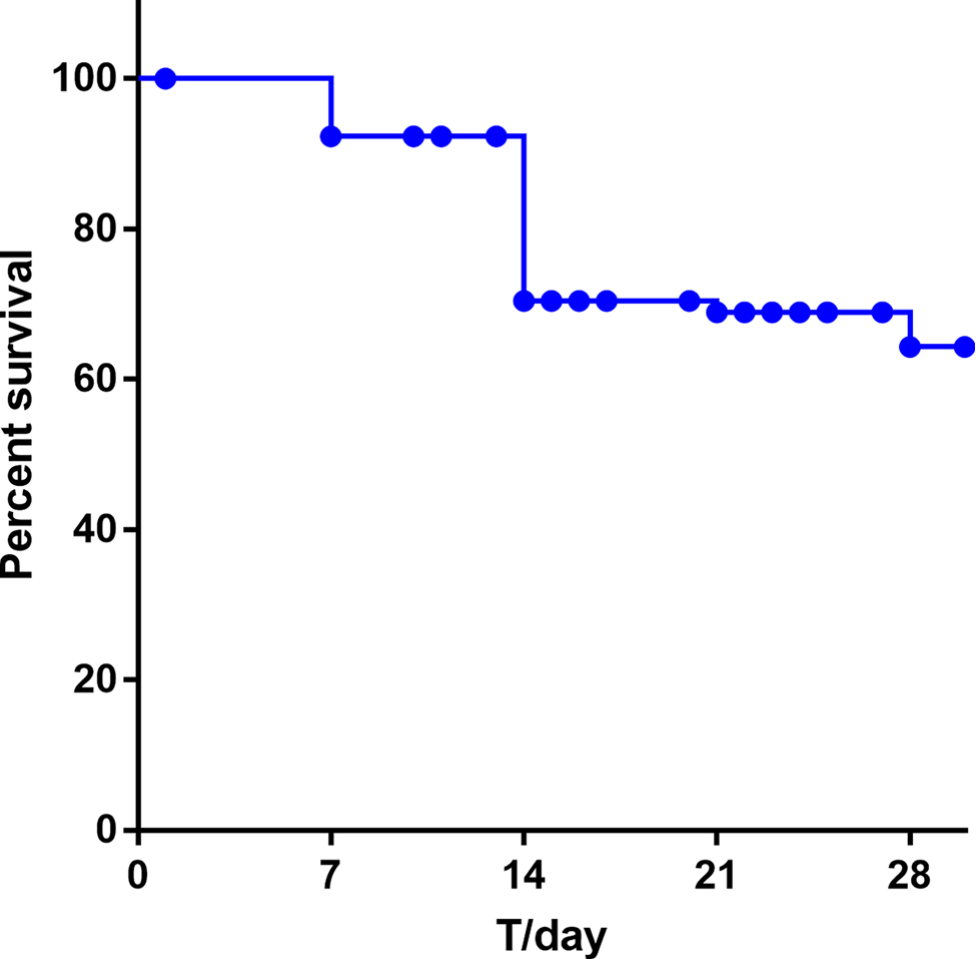
Neonatal mortality at 28 days with bacteremia.

We conducted a retrospective case–control study to evaluate the risk factors and clinical outcomes of the patients infected with sepsis from 2017 to 2019. Collectively, 91 sepsis cases were matched to 100 non-sepsis controls in our study. The prognosis of patients with sepsis was more likely to have stayed much longer in hospital than those from the control group, and they were more possibly to bead mission to ICU. The 14-day and 21-day survival rates for neonatal sepsis were 72.52% and 70.11%, respectively. Sepsis eventually caused 30.76% of newborn deaths and have the highest mortality rate during 7 to 14 days of hospitalization (**Figure 5**). In the multivariate analysis, patients with ICU admissions (OR, 15.91; 95% CI, 2.08–122.45; P = 0.008), low birth weight newborn (LBW; OR, 3.01; 95% CI, 2.02–4.14; P<0.001), very low birth weight newborn (VLBW; OR, 3.82; 95% CI, 1.72–5.92; P<0.001), extremely low birth weight newborn (ELBW; OR, 2.68; 95% CI, 1.04–4.32; P=0.001), premature delivery (OR, 2.11; 95% CI, 1.37–2.80; P<0.001), lower Apgar score (P<0.05), dyspnea (OR, 15.32; 95% CI, 2.38–98.67; P=0.04), Young Infants Clinical Signs Study (YICSS) (OR, 9.62; 95% CI, 1.42–64.98; P = 0.02), and their mother with antenatal corticosteroids (OR, 14.62; 95% CI, 1.07–198.49; P=0.04), chorionic inflammation (OR, 28.77; 95% CI, 2.16–382.64; *P* = 0.01) were more likely to suffer from sepsis (**Table 6**).

**Table 6:** Univariate and multivariate analyses of risk factors and outcomes for sepsis neonate compared with non-sepsis neonate from 2017 to 2019.

Variable	Sepsis Neonate (n=91)	Non-Sepsis Neonate (n=100)	Univariate logistic regression		Multivariate logistic regression	
			OR (95%CI)	P-value	OR (95%CI)	P-value
**Prognosis**						
Length of Hospitalization	23.69 ± 16.59	6.15 ± 208	NA	<0.001		
Admission to ICU	59.00 (64.83%)	6.00 (6.00%)	8.41 (2.38–o 29.67)	0.001	15.91 (2.08–122.45)	**0.008**
Length of ICU staying	13 ± 17.39	0.15 ± 0.64	NA	<0.001		
Dead	28.00 (30.76%)	NA	NA	NA		
**Neonatal characteristics**						
Male	55.00 (60.43%)	59.00 (59.00%)	0.89 (0.47–1.68)	0.74		
Age (>3 years)	51.00 (56.04%)	47.00 (47.00%)	1.21 (0.65–2.27)	0.53		
**Weight**						
LBW(<2500g)	38.00 (41.75%)	10.00 (10.00%)	21.74 (7.50–63.02)	<0.001	3.01 (2.02–4.14)	**<0.001**
VLBW(<1500g)	19.00 (20.08%)	4.00 (4.00%)	46.00 (5.63–375.38)	<0.001	3.82 (1.72–5.92)	**<0.001**
ELBW(<1000g)	7.00 (7.69%)	2.00 (2.00%)	14.63 (2.84–75.36)	0.001	2.68 (1.04–4.32)	**0.001**
Premature delivery	40.00 (43.95%)	25.00 (25.00%)	8.21 (3.94–17.15)	<0.001	2.11 (1.37–2.80)	**<0.001**
Super prematurity	29.00 (31.86%)	10.00 (10.00%)	0.57 (0.11–3.00)	0.51	0.56 (0.02–1.01)	0.43
IVF	13.00 (14.28%)	6.00 (6.00%)	5.77 (1.52–21.86)	0.01	1.50 (0.10–21.79)	0.76
**Apgar score**						
0min	9.17 ± 1.909	9.66 ± 1.07	NA	0.02		
5min	9.60 ± 1.14	9.92 ± 0.33	NA	0.007		
10min	9.62 ± 1.32	10.00 ± 0.00	NA	0.005		
Dyspnea	34.00 (37.36%)	8.00 (8.00%)	13.22 (5.23–33.37)	<0.001	15.32 (2.38–98.67)	0.04
Polyembryony	31.00 (34.06%)	18.00 (18.00%)	1.34 (0.56–3.21)	0.51		
Cesarean delivery	58.00 (63.73%)	41.00 (41.00%)	2.97 (1.31–6.73)	0.009	0.91 (0.14–5.91)	0.92
Premature rupture of membrane	45.00 (49.45%)	28.00 (28.00%)	2.54 (1.32–4.88)	0.005	3.83 (0.77–19.08)	0.10
YICSS	38.00 (41.75%)	6.00 (6.00%)	7.17 (2.21–23.36)	0.001	9.62 (1.42–64.98)	**0.02**
Digestive system disease	19.00 (20.87%)	18.00 (18.00%)	1.82 (0.88–3.85)	0.11		
**Treatment**						
Mechanical ventilation	31.00 (34.06%)	2.00 (2.00%)	21.31 (4.77–95.01)	<0.001	6.64 (0.30–146.43)	0.23
Deep vein catheterization	41.00 (45.05%)	35.00 (35.00%)	2.37 (0.39–18.97)	0.82		
CPAP	44.00 (48.35%)	34.00 (34.00%)	3.46 (0.32–37.25)	0.30		
Intubation tube	36.00 (39.56%)	3.00 (3.00%)	36.17 (10.07–129.94)	<0.001	1.96 (0.20–19.31)	0.56
**Characteristics of pregnant women**						
Antenatal corticosteroids	21.00 (23.07%)	3.00 (3.00%)	17.26 (1.65–180.24)	0.017	14.62 (1.07–198.49)	**0.04**
Chorionic inflammation	19.00 (20.87%)	1.00 (1.00%)	26.14 (2.98–228.89)	0.003	28.77 (2.16–382.64)	**0.01**
Gestational diabetes mellitus	19.00 (20.87%)	3.00 (3.00%)	9.09 (2.13–38.68)	0.003	8.68 (0.88–85.30)	0.06
Pregnancy-induced hypertension	6.00 (6.59%)	2.00 (2.00%)	1.04 (0.02–42.38)	0.98		
Intrahepatic cholestasis of pregnancy	2.00 (2.9%)	1.00 (1.00%)	3.11 (0.75–13.00)	0.12		
Hypothyroidism	5.00 (5.49%)	3.00 (3.00%)	5.16 (0.37–71.38)	0.22		

LBW, low birth weight newborn; VLBW, very low birth weight newborns; ELBW, extremely low weight newborn; IVF, in-vitro fertilization; YICSS, Young Infants Clinical Signs Study; CPAP, continuous positive airway pressure; NA, not applicable.

Bold values, P<0.05.

### Risk Factors for MDR Infection

We assessed the risk factors of sepsis with MDR and non-MDR (**Table 7**). As shown in the **Table 4**, Late-onset sepsis and antibiotic exposure were significantly associated with MDR infection (P<0.05).

**Table 7:** Clinical data analysis of neonatal infection with MDR from 2017 to 2019.

Variable	Total (n=91)	MDR (n=57)	Non-MDR (n=34)	P value
Male	56 (61.5%)	36 (63.1%)	20 (58.8%)	0.6
**Gestational age**				
Premature births	64 (70.3%)	40 (70.1%)	24 (70.6%)	0.9
Term delivery	27 (29.6%)	17 (29.9%)	10 (29.4%)	
**Weight**				
≤2,500 g	62 (68.1%)	39 (68.4%)	23 (67.6%)	0.8
>2,500 g	29 (31.9%)	18 (31.6%)	11 (32.4%)	
**Delivery mode**				
Caesarean section	58 (63.7%)	35 (61.4%)	23 (67.6%)	0.61
Natural labor	33 (36.3%)	22 (38.6%)	11 (32.4%)	
**Pathogens**				
*E. coli*	60 (64.5%)	38 (65.5%)	22 (62.9%)	0.79
*K. pneumoniae*	24 (25.8%)	15 (25.8%)	9 (25.7%)	0.98
Early onset sepsis	34 (37.3%)	16 (28.1%)	18 (52.9%)	**0.02**
Late-onset sepsis	57 (62.7%)	41 (71.9%)	16 (47.1%)	
**Inflammatory biomarkers**				
WBC (*10^9)	11.9 ± 7.6	11.9 ± 7.6	11.1 ± 8.49	0.5
N%	63.8 ± 19.4	64.7 ± 19.0	62.38 ± 20.1	0.6
PLT (*10^9)	200.3 ± 108.7	197.9 ± 120.7	204.4 ± 88.4	0.1
PDW	13.7 ± 3.7	13.73 ± 3.9	13.7 ± 3.4	0.4
I/T	23.9 ± 12.0	22.5 ± 11.1	26.3 ± 13.1	0.1
CRP (mg/L)	34.2 ± 27.2	27.4 ± 27.6	25.2 ± 25.3	0.2
PCT (ng/ml)	35.1 ± 41.9	46.8 ± 61.9	49.3 ± 56.7	0.8
Maternal infection	20 (21.9%)	13 (22.8%)	7 (20.5%)	0.7
Culture of Embryonic membrane	21 (23.1%)	13 (22.8%)	8 (23.5%)	0.9
Antibiotic exposure	18 (19.7%)	15 (26.3%)	3 (8.8%)	**0.03**

Bold values, P<0.05.

## Discussion

In the present study, we observed that sepsis pathogen distribution among the hospitalized neonates is changing significantly over the last two years, with noting that gram-negative bacteria emerged as the predominant causative organisms as well as more than half of sepsis patients carried MDR pathogens. In addition, this study deserves special attention for the following reasons.

First, we found two thirds of all sepsis occurred after 72 h, and the median age was 9 days. It contrasts starkly with one previous report, which stated most episodes of sepsis took place at the earliest possible age, with nearly one-quarter of culture-positive sepsis episodes occurring within 24 h of birth or two-thirds within 72 h (Investigators of the Delhi Neonatal Infection Study, 2016). Despite this, China (Al-Taiar et al., 2013; Xie et al., 2020) and Korea (Shim et al., 2011) have shown a predominance of late-onset sepsis. Interestingly, gram-negative bacteria, especially *Enterobacter*, such as *E. coli* and *K. pneumonia*, have become the predominant pathogens in different regions worldwide during the last two years (Sundaram et al., 2009; Tiskumara et al., 2009; Shim et al., 2011; Mintz et al., 2020; Xie et al., 2020), which were consist with our study. Other gram-negative bacilli, such as *Klebsiella aerogenes*, *Enterobacter cloacae*, and *Acinetobacter baumannii*, accounted for a very small proportion in the study, whereas other studies had found *Acinetobacter* spp. (22%) to be the most common pathogen in their cohort, which was usually rare at our hospitals (Investigators of the Delhi Neonatal Infection Study, 2016).

Second, we found that the pathogens associated with neonatal sepsis and their resistant patterns can differ between early-onset and late-onset sepsis. Remarkably, only about a third of *E. coli* in early-onset sepsis produced ESBLs, far less than *K. pneumonia* did in late-onset sepsis. A retrospective report from India stated that 61% (46/75) of neonatal gram-negative septicemias were ESBL-producing cases (Sehgal et al., 2007). In addition, a global investigation with a total of 71326 children in 30 different regional studies demonstrated that MDR was present in 30% of them in Asia and unexpectedly, 75% in Africa (Le Doare et al., 2015). A study from Nanjing, China, found that the proportion of MDRO infections was significantly higher among patients with late-onset sepsis than among those with early-onset sepsis (57.9% vs. 41.5%, *P* = 0.017) (Xie et al., 2020), which were consist with our result. In addition, we also detected the resistance mechanism among neonates infected with MDR pathogens, with all carbapenem-resistant bacteria carrying *bla*_NDM-1_, in accordance with other report in China (Yan et al., 2017). Moreover, we found that nearly all the strains carried CTX-M-15, more than half of them harbored SHV-1 and TEM, which was quite different from Taiwan with the SHV-type ESBLs the most prevalent among patients (Tsai et al., 2016). However, data from India (Roy et al., 2015), South America (Logan et al., 2014) and Peru (Garcia et al., 2016) showed CTX-M-15 the most commonly detected ESBLs. In addition, we also found that qnrB mediated resistant to quinolone were popular among pathogen, whereas AmpC genes and aminoglycoside resistance genes were much rarer, which were consist with the researches in China (Sehgal et al., 2007; Jiang et al., 2008). Therefore, setting up guidelines for antibiotic use in neonatal sepsis based on countries should be an urgent priority to prevent their further spread.

Third, our study had shown that the case fatality rate was 30.76% for culture-positive sepsis cases, which is similar to sepsis in India (Investigators of the Delhi Neonatal Infection Study, 2016). However, the overall 30-day all-cause mortality in the group of patients with BSI in south-east Sweden was 13%, which might be related to the low rate of bacterial resistance in Sweden (Holmbom et al., 2020). In light of sepsis showing the longer hospitalization and higher mortality, our study explored for the first time the independent risk factors of sepsis among neonatal sepsis. Our results demonstrated that ICU admissions, LBW, VLBW, ELBW, premature delivery, lower Apgar score, dyspnea, YICSS, and their mother with antenatal corticosteroids, chorionic inflammation were identified as independent risk factors to acquisition of sepsis. Despite the high mortality rate in neonates with sepsis, few recent studies have examined the incidence of this severe neonatal infection. WHO Young Infants Clinical Signs Study (YICSS) had defined possible severe bacterial infection (including sepsis, meningitis, and pneumonia) as the presence of any one of a history of difficulty feeding, history of convulsions, movement only when stimulated, respiratory rate of 60 or more breaths per min, severe chest indrawing, and a temperature of 37.5°C or higher or 35.5°C or lower (Young Infants Clinical Signs Study, 2008). In some cases, using this standard will result in missing the diagnosis or misdiagnosing sepsis. Our findings were supplemented to help develop new disease prediction models and policy in support of the post-2015 agenda to end preventable child deaths.

Finally, we found late-onset sepsis and antibiotic exposure were significantly associated with MDR infection. Ampicillin or cephalosporin is often used as an empirical treatment for bacteremia, but these drugs may cause a high expression of AmpC and ESBLs, leading to resistance to β-lactam antibiotics (Holsen et al., 2019). Piperacillin-tazobactam (TZP), a weak inducer of AmpC beta-lactamases and inhibitor of ESBLs, may be a valuable treatment option for sepsis (Cheng et al., 2017).

## Conclusion

Our study showed that sepsis was a serious emerging challenge among hospitalized neonates. Gram-negative bacteria gradually became the main pathogenic bacteria of neonatal sepsis, with *E. coli* and *K. pneumoniae* contributing the largest proportion. Moreover, late-onset sepsis and antibiotic exposure were significantly associated with MDR infection. Other antibiotics, such as TZP, should be considered as the first choice in empirical antimicrobial therapy.

## Data Availability Statement

The original contributions presented in the study are included in the article/supplementary material. Further inquiries can be directed to the corresponding authors.

## Ethics Statement

The studies involving human participants were reviewed and approved by Chongqing Health Center for Women and Children. Written informed consent to participate in this study was provided by the participants’ legal guardian/next of kin.

## Author Contributions

HZ and XH designed the study. LG and AL offered the data. CL and YShen performed the experiments. QH and YSu analyzed data. HZ and XJ wrote this manuscript. All authors contributed to the article and approved the submitted version.

## Funding

This study was supported in part by the Science and Technology Research Program of Chongqing Municipal Education Commission (Grant No. 2021MSXM090).

## Conflict of Interest

The authors declare that the research was conducted in the absence of any commercial or financial relationships that could be construed as a potential conflict of interest.
